# Habitat adaptation rather than genetic distance correlates with female preference in fire salamanders (*Salamandra salamandra*)

**DOI:** 10.1186/1742-9994-6-13

**Published:** 2009-06-29

**Authors:** Barbara A Caspers, Claudia Junge, Markus Weitere, Sebastian Steinfartz

**Affiliations:** 1 University of Bielefeld, Dept. Behavioural Biology, Research Group of Molecular Ecology and Behaviour, Morgenbreede 45, D-33615 Bielefeld, Germany; 2 Centre for Ecological and Evolutionary Synthesis (CEES), Dept. of Biology, University of Oslo, PO Box 1066, N-0316 Oslo, Norway; 3 University of Cologne, Zoological Institute, Weyertal 119, D-50923 Cologne, Germany

## Abstract

**Background:**

Although some mechanisms of habitat adaptation of conspecific populations have been recently elucidated, the evolution of female preference has rarely been addressed as a force driving habitat adaptation in natural settings. Habitat adaptation of fire salamanders (*Salamandra salamandra*), as found in Middle Europe (Germany), can be framed in an explicit phylogeographic framework that allows for the evolution of habitat adaptation between distinct populations to be traced. Typically, females of *S. salamandra *only deposit their larvae in small permanent streams. However, some populations of the western post-glacial recolonization lineage use small temporary ponds as larval habitats. Pond larvae display several habitat-specific adaptations that are absent in stream-adapted larvae. We conducted mate preference tests with females from three distinct German populations in order to determine the influence of habitat adaptation versus neutral genetic distance on female mate choice. Two populations that we tested belong to the western post-glacial recolonization group, but are adapted to either stream or pond habitats. The third population is adapted to streams but represents the eastern recolonization lineage.

**Results:**

Despite large genetic distances with F_ST _values around 0.5, the stream-adapted females preferred males from the same habitat type regardless of genetic distance. Conversely, pond-adapted females did not prefer males from their own population when compared to stream-adapted individuals of either lineage.

**Conclusion:**

A comparative analysis of our data showed that habitat adaptation rather than neutral genetic distance correlates with female preference in these salamanders, and that habitat-dependent female preference of a specific pond-reproducing population may have been lost during adaptation to the novel environmental conditions of ponds.

## Background

A crucial step in ecologically driven population differentiation and potential speciation is the adaptation of recently diverged sub-populations to new habitats [1,2]. Although habitat-dependent divergence can be opposed by migration between differentially adapted subpopulations [3], reproductive isolation can be achieved either by strong natural selection against immigrants from divergent habitats [4,5] or by the evolution of mate preferences. Such preferences will reinforce the genetic split between differentially adapted sub-populations, eventually leading to a speciation event [2,6-9]. The roles of recent habitat adaptation and consequent divergent selection on the evolution of mate preferences are widely recognized in the literature [1], and have been demonstrated in laboratory settings [10,11]. However, comprehensive studies integrating ecological, molecular and experimental behavior approaches showing changes in mate preference correlated with recent habitat adaptations are rare. The most common system to be studied from this perspective is the three spine stickleback that recolonized many freshwater lakes of the Northern Hemisphere after the last glaciation and has, according to different habitat use, adapted into different reproductively isolated benthic (feeding on the lakebed) and limnetic (feeding on plankton) species that mate assortatively [12-14].

By integrating molecular, ecological and demographic approaches [15-19], we have developed another promising natural system that utilizes an amphibian species, the fire salamander (*Salamandra salamandra*, Linnaeus 1758). We have studied this species in detail to describe the processes and effects of recent habitat differentiation in a vertebrate population. In the current paper, we integrate behavioral data of *S. salamandra *within an explicit phylogeographic framework in order to describe the extent to which habitat adaptation and genetic distance between populations influences female mate preference.

**Figure 1:**
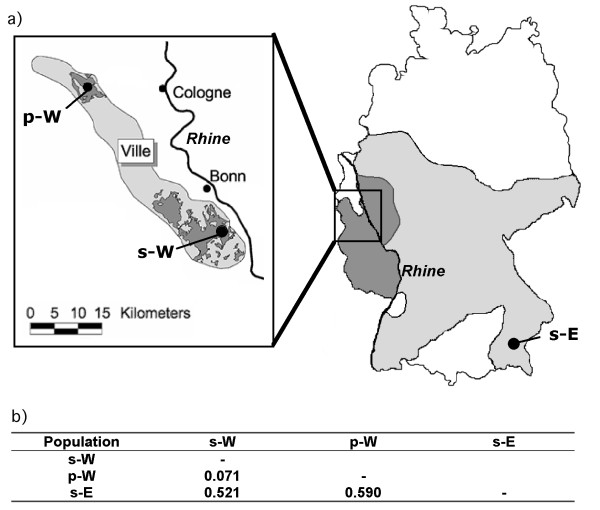
**(a): Locations of *Salamandra salamandra *populations in Germany from which adult salamanders were collected to be used for mate preference tests and microsatellite loci analysis**. Light grey indicates the distribution of salamander populations belonging to the eastern post-glacial recolonization lineage; dark grey denotes the distribution of populations from the western post-glacial recolonization lineage in Germany (after [16]). We analysed individuals from one pond-adapted (p-W) and one stream-adapted (s-W) population within the western post-glacial recolonization lineage and from one stream-adapted population within the eastern post-glacial recolonization lineage (s-E). **(b): **Table of neutral genetic differentiation (expressed as Reynolds' F_ST _values) based on nine microsatellite loci analyzed between populations (see *Genetic analysis *of Methods for details). All pairwise population comparisons are significant on a 5% level.

Fire salamanders recolonized Germany through two independent lineages, one eastern and one western, after the end of the last European glaciation event, roughly 8,000 years ago [[15,16,20], see Fig. 1a]. Typically, females of *S. salamandra *deposit their larvae in small permanent streams. However, as a habitat-dependent adaptation, small temporary ponds are also used as larval habitats by the western recolonization lineage [16,21,22]. Larvae developing in ponds display several habitat-specific adaptations absent in stream larvae, including a greater larval weight at birth, the ability to thrive on lower quality sources of food, and early metamorphosis under conditions of limited food conditions [16]. Adaptations to stream reproduction may be considered the ancestral condition for *S. salamandra *in Middle Europe, whereas adaptation to pond reproduction is a post-glacially derived adaptation of certain populations of the western recolonization lineage [[16], see Fig. 1a). After metamorphosis occurs in the respective larval habitats, the juvenile and adult life stages of the fire salamander are strictly terrestrial. Females mate with multiple males [23], and mating is not associated with larval habitats [22].

It is still generally thought that geographic distance results in greater neutral genetic divergence, which in turn increases the diversification of mate recognition mechanisms and associated signals [[24-27], but see [28] for a more complex and critical view]. In *S. salamandra*, such mate recognition signals may be communicated by olfactory cues, as is found in other salamanders [29-31]. Indeed, recent behavioral studies showed that closely related species (i.e., *S. atra *and *S. lanzai *[32,33] and *Lyciasalamandra *[34]) use odor cues for different kinds of intraspecific communication. We hypothesize that the olfactory system of *S. salamandra *is involved in mate recognition, and in this study we used odor preference tests to investigate the preference of female salamanders for males.

The major aim of this study was to test whether the preferences that females displayed in the odor tests correlated with reproductive adaptation to either stream or pond environments, or to the neutral genetic distance measured by divergence of nuclear microsatellite loci.

## Results

**Figure 2:**
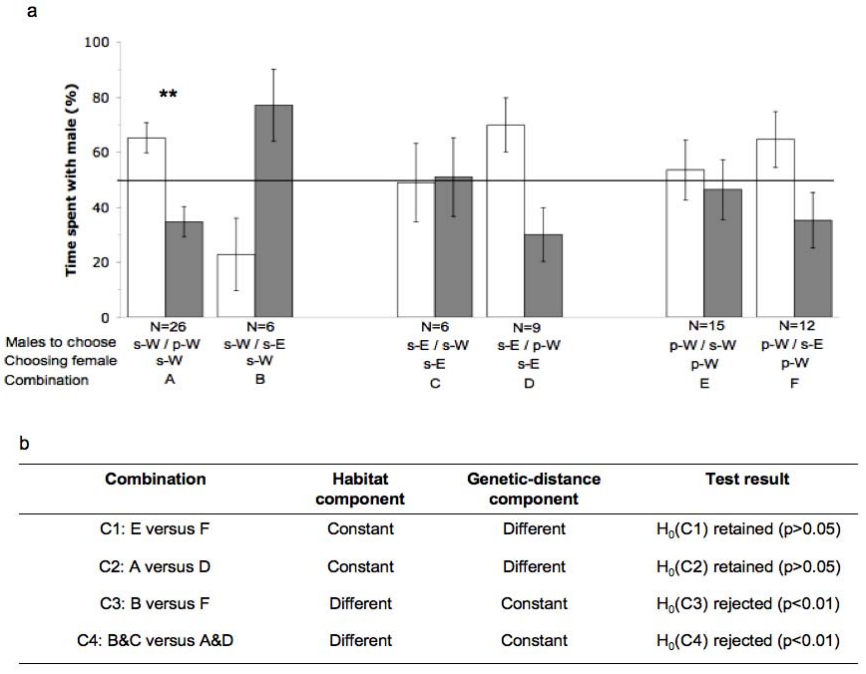
**Combination of female preference tests for males in *Salamandra salamandra***. **(a) **Six different female choice trial combinations (A-F) were performed. In each combination, tested animals were characterized by their habitat adaptation to streams (prefix *s*) or to ponds (prefix *p*), and by their origin of the western (W) or eastern (E) post-glacial recolonization lineage. In each combination, a female could choose between a male from its own population and a male from a foreign population. The amount of time spent by a female with each male is shown, expressed as percent of total time spent in each of the two peripheral areas. Bars represent means; error bars represent standard error. One-factorial ANOVA revealed significant (p < 0.05) effects of different combinations. **(b) **Specific combinations (C1-C4) of choice trials (A-F in (a)) that were tested against each other to determine whether the habitat component (i.e., habitat adaptation to streams or ponds) or the genetic distance component (i.e., the neutral genetic divergence inferred from microsatellite differentiation) influenced female mate preference with the null hypothesis that females spent the same amount of time with their own male. In each combination, either the habitat or the genetic distance component was kept constant. For further details see section *Data analysis of female preference tests*.

Females spent significantly different amounts of time with males of their own population depending on the combination of traits in the other male (A-F; Fig. 2b; ANOVA: F_5,68 _= 3.304, p = 0.046), indicating that female mate choice has been influenced either by habitat or by genetic distance. When paired with males from the pond habitat (p-W; combination A in Fig. 2a), stream-adapted females of the western recolonization lineage (s-W females) spent significantly more time with males from their own population (151.5 ± 16.3 min [65.3 ± 5.5%]; one-sample t-test: T_25 _= 2.79; p < 0.01). However, the opposite trend (43.3 ± 21.2 min [22.9 ± 13.2%]; one-sample t-test: T_5 _= -2.06; p = 0.094) was observed if they were given access to stream-adapted males from the eastern post-glacial recolonization lineage (population s-E; combination B in Fig. 2a). Females from s-E did not show any preference for their own males when compared to stream males of the other post-glacial recolonization lineage (s-W; 95.0 ± 32.8 min [48.9 ± 14.3%]; one-sample t-test: T_5 _= -0.07; p = 0.95 combination C in Fig. 2a). However, they showed a slight, non-significant preference for males from their own population (160.3 ± 36.0 min [69.9 ± 9.8%] one-sample t-test: T_8 _= 2.04; p = 0.076) compared to males from pond habitats (p-W; combination D in Fig. 2a). No significant preference was recorded for pond-reproducing females (p-W) when given stream males representing their own (s-W; 141.4 ± 32.6 min [53.6 ± 10.9%]; one-sample t-test: T_14 _= 0.33; p = 0.747; combination E in Fig. 2a) or the other post-glacial recolonization lineage (145.3 ± 31.9 min [64.7 ± 10.2%]; one-sample t-test: T_11 _= 1.45; p = 0.175; combination F in Fig. 2a).

When tested for significant deviations from Hardy-Weinberg equilibrium, only locus Sal 29 in population s-E (Nasenbach) showed a significant deviation (p = 0.001), whereas all other comparisons were in Hardy-Weinberg equilibrium. All pairwise population comparisons based on nine microsatellite loci were significant on a 5% level. As expected from the post-glacial population history of *S. salamandra *in Middle Europe, nuclear genetic distance (F_ST_) between populations of the different post-glacial recolonization lineages based on putative neutrally evolving microsatellite loci ranged between 0.522 (s-E/s-W) and 0.590 (s-E/p-W), whereas the F_ST _distance between populations s-W and p-W of the western recolonization lineage was nearly 10-fold lower (F_ST _= 0.071).

For the tested combinations in which the habitat component was constant and the genetic distance component was different (C1: E versus F and C2: A versus D; see Fig. 2a,b), the null hypotheses that females spent the same amount of time with males from their own population could not be rejected (α = 0.05). However, for both combinations in which the genetic distance component was constant and the habitat component differed between choice trials (C3: B versus F and C4: B&C versus A&D; see Fig. 2a,b), the null hypothesis that females spent the same amount of time with males from their own population was rejected (α = 0.01).

## Discussion

It is widely accepted that adaptation to new habitats can drive population differentiation and speciation through reproductive isolation. In this context, female preference for males from the same habitat type is interpreted as habitat dependent assortative mating. In three-spine sticklebacks, it has been demonstrated that differences in body size are coupled with ecological differentiation (i.e., benthic species feeding on the lakebed and limnetic species feeding on plankton), and that females use this characteristic to recognize and mate assortatively according to habitat [13,14]. German populations of the fire salamander (*Salamandra salamandra*) have adapted to different larval habitats (i.e., to streams or small temporary ponds), where larvae develop until metamorphosis. It has previously been ambiguous whether female mate preference in *S. salamandra *is correlated with the habitat type as mating in *S. salamandra *is strictly terrestrial and not associated with the habitat where females will deposit larvae. Since olfactory cues are known to be involved in intraspecific communication in terrestrial salamanders [29-34], we searched for olfactory cues as possible mate recognition signals in the fire salamander. It is generally thought that geographic distance results in greater neutral genetic divergence, which in turn increases the diversification of intraspecific mate recognition mechanisms and associated signals [26,28]. The present study, however, indicates that it is habitat adaptation rather than neutral genetic distance that influences female mate preferences in the fire salamander.

Fire salamanders recolonized Germany through both eastern and western recolonization lineages after the last glaciation, and different lineages are associated with diagnostic mitochondrial haplotypes [16]. Our study setup includes one population (s-E) from the eastern recolonization lineage, and two geographically close populations from the western recolonization lineage (populations s-W and p-W). As expected from the post-glacial recolonization history and the mitochondrial data, our microsatellite analysis supports a strong genetic differentiation between these two lineages. Population s-W and p-W, which belong to the same recolonization lineage, showed a small but significant amount of genetic differentiation (F_ST _= 0.071). The neutral genetic divergence between populations of the different recolonization lineages was nearly 10-fold higher (F_ST_(s-E/s-W) = 0.523 and F_ST_(s-E/p-W) = 0.590). Interestingly, adaptation to reproduction in ponds seems to be specific for the western recolonization lineage, and is thought to be a post-glacial adaptation [16]. The fact that it has been not observed so far in the eastern recolonization lineage does not completely rule out that such an adaptation could have also evolved in this lineage. Female choice trials reflected a situation in which a female could choose a male from her own population (representing identical habitat and genetic distance component) and a foreign male that differed in at least one of these respects.

We found a clear trend to indicate that stream-adapted females of both lineages discriminated against pond-adapted males (combination A (p < 0.01) and D (p = 0.076), Fig. 2a). Despite the small amount of genetic divergence between individuals of s-W and p-W, s-W females significantly preferred males of their own population if they had to choose between those and pond-adapted males from the same recolonization lineage (combination A in Fig. 2a; p < 0.01). The opposite (though non-significant) trend was observed in combination B (n = 6); here s-W females could choose between males from their own population and males from s-E that differed in the genetic distance component but not in the habitat component. This trend might become even a significant difference if the sample size were increased. In contrast, pond-adapted females did not show any preference for their males from their native population over stream-adapted males (combination E and F, Fig. 2a). Pairwise comparisons of choice trial combinations revealed that the genetic distance component did not impact female preference, but that habitat adaptation significantly impacted female preference (p < 0.01; Fig. 2b).

Although our data support the hypothesis that habitat adaptation rather than genetic distance influences female mate preference, our results also show that adaptation to reproduction in ponds did not induce specific mating preferences in salamanders from this specific population. From our phylogeographical-ecological analysis, we can infer that pond reproduction evolved as a post-glacial adaptation to new environmental conditions, a situation that has thus far only been described in the western recolonization lineage. Reproduction in streams, however, must have been the ancestral condition [16]. As stream-adapted females of both recolonization lineages preferred males from the same habitat type but pond-adapted females did not, pond-adapted females of the western recolonization lineage might have lost their habitat-dependent preference during adaptation to the new environmental conditions of ponds.

In this context, an olfactory mate recognition study for a sympatric species pair of benthic and limnetic three-spine sticklebacks showed that benthic females preferred conspecific males, whereas limnetic females showed no preference for limnetic males [35]. However, as mentioned above, benthic and limnetic species are known to mate according to size [14]. It has been hypothesized that the ecological conditions experienced by the different species are responsible for differences in the recognition of mating signals, including body size and olfactory cues [35]. In our case mating of salamanders is strictly terrestrial and the habitat occupied by the adults used in this study does not contain observable environmental differences, as is the case in the three-spine stickleback system. Therefore it is unlikely that different habitat conditions influence the perception of mating cues in adults. On the other hand, a lack of mating preferences according to habitat-dependent morphs (paedomorphic *versus *metamorphic) has been described for the Alpine newt (*Mesotriton alpestris*; [36]), as in this system facultative environmentally induced paedomorphosis seems to be determined by different ontogenetic pathways in a specific individual [37].

It is difficult to determine whether pond adaptation in *S. salamandra *is coupled with female preferences for males of the same habitat type from our present study. However, such mating preferences must be present when pond and stream adapted salamanders occur under sympatric conditions; otherwise, the observed genetic differentiation in such situations would not be maintained (see [17] for details and discussion of this point). Future mate choice experiments will therefore focus on natural contact situations of both types to explore to which degree female preference has evolved. In parallel, we aim to increase the number of replicate populations of both habitat types in order to generalize our hypothesis of habitat-dependent evolution of mating preferences in *Salamandra salamandra*.

## Conclusion

Our data generally support the hypothesis that habitat adaptation rather than neutral genetic distance influences female mate preference in *S. salamandra*. Our results highlight the importance of comprehensive studies integrating ecological, molecular and experimental behavior approaches to understand the interaction of divergence in mate preference as a consequence of habitat adaptations, which can be a first and crucial step during the process of speciation.

## Methods

### Study design and test setup

The adult salamanders used in this study originated from one pond-adapted (located in Königsdorf 50°56'24 N, 6°44'48 E; 130 m a.s.l. hereafter abbreviated as p-W) and one stream-adapted (located in the Kottenforst; 50°41'09 N, 7°07'03 E; 180 m a.s.l. hereafter abbreviated as s-W) population of the western post-glacial recolonization lineage of *S. salamandra *in Germany. Salamanders from Bavaria represented the stream-adapted population (located in Nasenbach 48°11'35 N, 12°27'35 E; 438 m a.s.l. hereafter abbreviated as s-E) of the eastern post-glacial recolonization lineage (see Fig. 1a for geographic distribution of recolonization lineages and populations). All salamanders were collected from the field, maintained for the time of the behavioral experiments and sampled for DNA analysis with respective permissions from the "Untere Landschaftsbehörde der Stadt Bonn" and the "Regierung von Oberbayern". Maintenance of salamanders as well as the experimental setup was supervised by the Tierschutzbeaufragter of the University of Bielefeld, Dr. Schmitz.

Fire salamanders in Middle Europe usually deposit larvae that were fertilized in the preceding reproductive period in the oviduct of the female (see [23] for details) at the onset of early spring. Salamanders of both sexes were collected during the onset of the reproductive season in March 2006, and most females were collected during the deposition of larvae into or when immediately approaching the respective reproduction habitats. Males were collected from an area not more than 100 m away from the respective reproduction habitat. Given that home range size of *S. salamandra *in a very similar habitat ranged between 78 – 2266 m^2 ^[19] and other reproduction habitats were several kilometers away in our setup, our collection approach should definitely include females and males that are associated with the respective reproduction habitat. As mating in *S. salamandra *under natural conditions occurs after the deposition of larvae [22], we assumed that when we collected them, females had not yet mated during this reproductive season and were therefore receptive. After capture, each individual was kept separately in a box-like terrarium (length = 41 cm; height = 14 cm; width = 18 cm), and only came in contact with other salamanders during the odor preference tests. Each box was fitted with the same kind of substances: a five cm ground layer composed of soil and clay that was covered with pieces of wood from the forest to provide shelter for the individual. All salamanders were fed once a week with either earthworms or crickets. Boxes with salamanders were kept in climate controlled chambers with day-night cycles of 12 hours, consisting of fluorescent light (Osram L 58 W lumilux daylight) and 18°C constant temperature followed by complete darkness and 16°C temperature.

**Figure 3:**
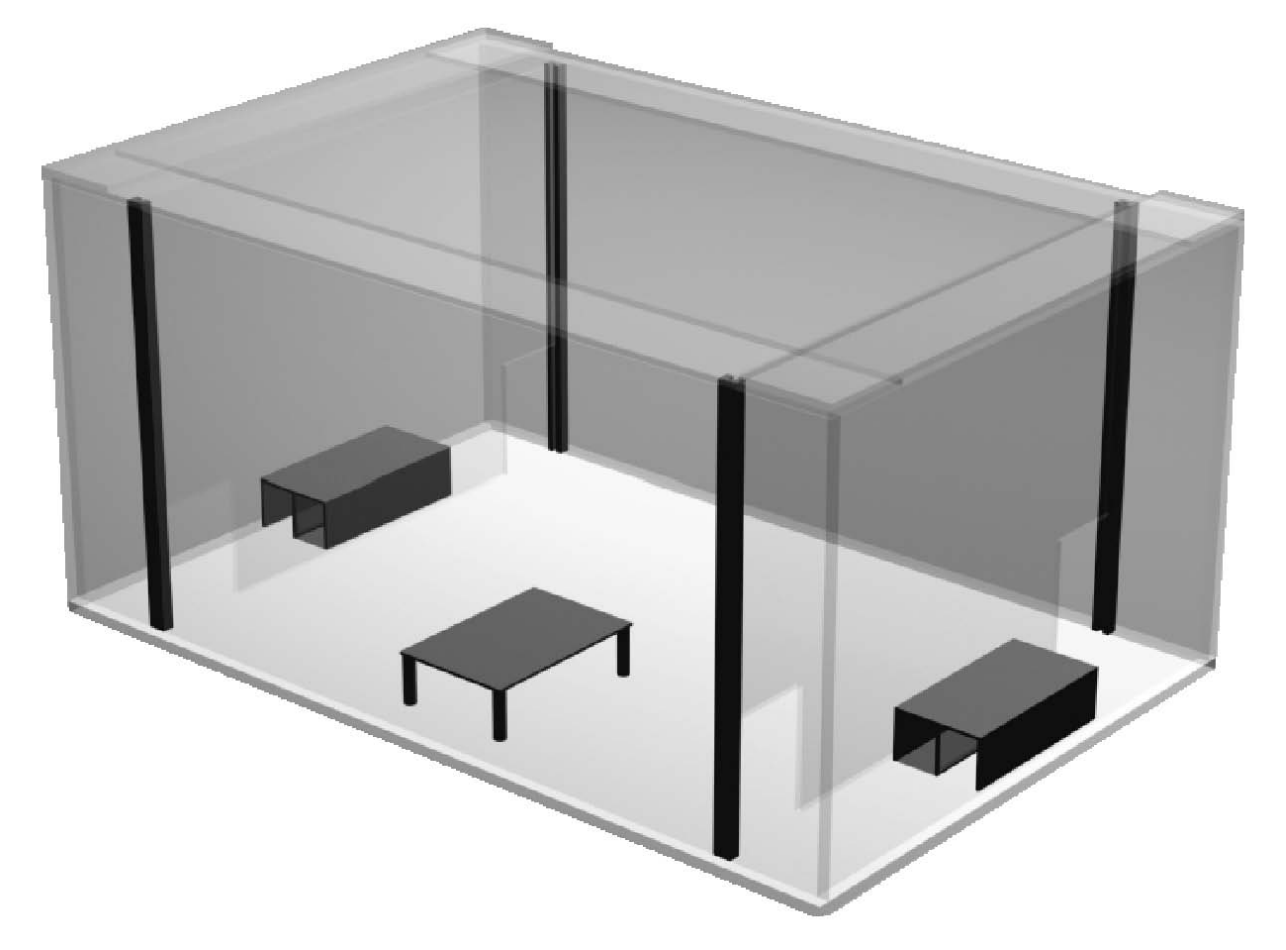
**Diagram of setup for female preference tests (see *Study design and test setup *of Methods for details)**. Females were placed in the central compartment at the beginning of each trial. During a 10-hour time period, we measured the time females spent in each peripheral area, which were each implanted with the odor of a different male.

In total, 136 individuals (74 females and 62 males) were used in the female preference tests. All preference tests simulated possible contact situations in which a female could choose between a male from the same population (representing the identical habitat and a closely-related genetic type), and a male from a different population differing in one or both respects (see combination trials A-F in Fig. 2). Since under natural conditions salamanders are mainly active during the night, we performed preference tests during the 12-hour dark period. The test box was divided into a central and two peripheral areas (see Fig. 3 for details) and completely covered with tissue paper, which we moistened with water prior the test. At the beginning of the experiment, peripheral areas were separated from the central area with solid Plexiglas panes. A single male was placed for one hour into each peripheral compartment to pre-impregnate the compartment. During this period, males were allowed to move freely in their respective compartments. Subsequently, the males were placed in an air-permeable shelter, allowing diffusion of volatiles into the corresponding peripheral compartment (see Fig. 3). We replaced the solid Plexiglas panes by panes that allowed the female to enter peripheral compartments through small entrances in the bottom corners (see Fig. 3). At this point, a single female was placed under a shelter at a consistent center point of the central compartment, in order to control for differences in the starting positions of different females between tests. The activity of the females was recorded continuously under red light conditions (1–1.5 lx, PF 712E*B5, Philips, Germany) using highly photosensitive cameras (CCD black/white mini camera C3172, ELV Elektronik, Germany) for 10 hours. Each female was tested only once and some of the males up to three times for the preference tests. We washed the complete experimental setup with odorless soap (Praecutan ^®^, Degussa, Krefeld, Germany) after each trial and let it dry before performing the next trial.

### Data analysis of female preference tests

Using the focal sample method [38] we analyzed the ten-hour video tapes and measured the time females spent in each of the peripheral compartments within the second five-hour period, if females had visited both peripheral compartments within the first five-hour period before. This way we ensured that females had potentially perceived both stimulus odors. Analyzing persons were blind to population origin of males and the female in each trial. We calculated the ratio of time females spent with the male from the same population to the total time the female spent in both peripheral compartments.

Our statistical analysis addressed two questions: i) Do females prefer males from their own population, i.e., did they spend significantly more time with the male from their own population than with the other male? We analyzed six different choice trial combinations (A-F in Fig. 2a), in which females always could decide between a male from their own population and a male from a foreign population differing either in larval habitat, or genetic proximity, or both. Each choice trial combination was analyzed by a one-sample t-test for significant deviations from an equal distribution (50%) of time spent in each compartment. ii) Does female preference correlate with genetic distance (expressed as F_ST _distance based on microsatellite loci differentiation between populations of the western and eastern recolonization lineages; hereafter the genetic distance component) or with the shared habitat adaptation (i.e., adaptation to pond or stream reproduction; hereafter the habitat component)? In order to determine which component had a stronger impact on female mate preference, we tested specific combinations of choice trials so that either the genetic distance component or the habitat component was constant. In each of these four tests (see combinations C1- C4 in Fig. 2b) the null hypothesis was that females spent the same amount of time with their own male.

After using one-factorial ANOVA to reject the null hypothesis that the females spend equal amounts of time with males of their own population in choice trial combinations A-F, *a priori *tests for multiple comparisons were performed to test combinations C1-C4 for significant differences. The pairwise comparisons for combinations C1-C3 were performed with an LSD-test. The linear contrast = μ_C _+ μ_B _- μ_D _- μ_A _was tested for combination C4. The data were log (x + 1) - transformed before performing all analyses in order to achieve consistent variances.

### Genetic analysis

Genomic DNA was extracted from tissue samples (toe clips) of the same individuals that were used in the preference tests following published procedures [15]. Accordingly, 53 individuals were analyzed from Königsdorf (p-W), 55 individuals from the Kottenforst (s-W) and 28 individuals from the Nasenbach population (s-E). In order to test whether the habitat component or the genetic distance component (see section *(a) *for definitions) predominantly influenced female preference between populations, we determined the neutral genetic distance between these populations based on differentiation of nine microsatellite loci (locus Sal 3, Sal 29, Sal E2, Sal E5, Sal E6, Sal E7, Sal E8, Sal E11 and Sal E12). Amplification of loci, scoring of alleles and loci characteristics are described in [39]. Each population was tested for deviations from Hardy-Weinberg Equilibrium and received p-values were corrected for multiple comparisons. Population pairwise F_ST _values (Reynolds' F_ST_) were inferred and pairwise differences were tested for significance (α = 0.05). All analyses were done by using the Arlequin program (version 2.0 [40]).

## Competing interests

The authors declare that they have no competing interests.

## Authors' contributions

Conceived and designed the experiments: SS, MW. Performed the experiments: BC, CJ. Analyzed the data: BC, MW, CJ, SS. Designed figures: CJ, BC. Wrote the paper: SS, BC, MW. All authors read and approved the final manuscript.
